# Annotations

**Published:** 1899-04-22

**Authors:** 


### Annotations.

**The Limitation of Families in County Council Dwellings.**

The London County Council is finding out, and by no means for the first time in its history, how impossible it is to please all parties. Having gone, as some people would have it, considerably out of its way, and having greatly violated the canons of the economists, in building workmen's dwellings, the Council is now being abused for enforcing the wholesome regulations which it has framed with the object of preventing overcrowding in the tenements it has set up. We are being told, in fact, that these regulations are absolutely immoral, and that their direct effect is to encourage the abortion monger and to put a premium upon infanticide, and this all because the County Council will not allow in its workmen's dwellings a greater density of population than two to a room! A correspondent in the *Times* says. "What a farce it is to imprison the Dr. Collins's and Nurse Whites, and then allow the London County Council to say, 'You dare to have another child, and we put you in the street.' Can any greater inducement be given to kill their children before or after birth, especially when the difficulty of finding accommodation is so great as it

ANNOTATIONS—*continued.*

is now?" And so it is said that if the County Council is bent upon carrying out "this policy"—that is, the policy of preventing overcrowding—it is "morally bound" to provide still larger workmen's dwellings. Now as to the size of dwelling which the County Council will ultimately have to provide we express no opinion, although it seems clear enough that, if the authorities are going to drive away all private enterprise from the building of houses for the working classes, they must be prepared themselves to build houses of all sizes. That, however, is a question of policy, which we do not touch. But we do urge that wherever public money is used for the erection of such houses the sanitary standard aimed at shall be high. The building and the letting of workmen's dwellings by sanitary authorities involve great responsibilities which must not be shirked, and first among them is that of setting a standard of living and of sanitation. Authorities among whose proper functions is that of compelling unwilling landlords to bring their properties up to a certain standard of health and decency—a standard which it is to be hoped will rise year by year—cannot afford to be themselves the landlords of property which goes below or even fringes on the standard which they have to enforce on others by arm of law. We admit the hardship of large families, and the risk, we had almost said the wrong, of attempting to bring them up in the centre of great cities; and if the County Council, or any other public body which chooses to enter upon the business of house letting, will undertake to provide for these large families, well and good. But we protest against pressure being brought upon them to permit overcrowding in the houses they put up, and thus to perpetuate the very evils the suppression of which was the main excuse for allowing them to enter upon the trade of housebuilders for the people.

### Naked Lights.

The promise which is being held out of an incandescent electric light without the usual vacuum bulb is a matter of great interest from a sanitary point of view. Notwithstanding the numerous and manifold advantages offered by the electric light, the loss of the purifying effect of the open flame has always been recognised by some sanitarians, although not by all, as a distinct evil. No doubt the so-called "burning-up" of the air by gas or oil lamps is an evil; but at least it has this countervailing advantage, that the obvious heat and oppressiveness so produced lead of necessity to ventilation, whereas in a room lighted by electricity there is nothing to compel ventilation until the air is rendered more or less fetid by accumulated animal exhalations. But besides compelling ventilation the open flame has a great purifying influence, in that it actually burns up all the organic dust which passes through it, and the amount of air which passes through a gas-flame lamp in the course of an hour is very considerable. Years ago Professor Tyndall, when experimenting upon dust in its relation to disease, showed that one of the best ways of rendering the atmosphere in his experimental box free from germs was by enclosing in it a naked platinum wire, which was kept at a red heat by an electric current. Every particle of air was thus in turn brought into contact with the incandescent wire, and the dust it carried was consumed; and in just the same way our gas flames and oil lamps, although they use up some oxygen in the process, act as germ destroyers. If the Nernst electric light should turn out a success, its naked incandescent rod would no doubt give us back again the sanitary advantage of which we have been deprived while using the ordinary bulb light which has so largely occupied the field. The air-purifying properties of the arc light are believed to be still more marked, depending as they do, not on the heat alone, but on the constant evolution of ozone which goes on.

### A Picture in Bumbledom.

Lest people should imagine that when certain specific duties are laid by law upon certain legally appointed bodies they are sure to be properly carried out, it may be worth while to refer to the fact that for some time back the Oldham Workhouse has been in possession of a bailiff, and that the funds of the same union, to the tune of upwards of £17,000, have been held under a writ of sequestration in the hands of the High Court, owing to the refusal of the guardians to pay certain increases of salary ordered by the Local Government Board. The matter in dispute is no great thing, merely a few pounds of increase in the salary of some poor-rate collectors, but the incident is none the less important in view of the present tendency towards devolution and the generally expressed desire to give enlarged powers to local authorities. The Local Government Board may be slow and somewhat ponderous in its movements, but what is clear is that if local government is to work well a very strong central Board is necessary for the purpose of controlling our local governors. The Oldham workhouse is now, we believe, free from the presence of the "man in possession," Mr. Justice Channell, before whom the case came, having ordered the payment of the debt due to the collectors out of the sum in the hands of the High Court, and having released £10,000 of the same money for the use of the guardians in carrying on the work of the union. So the tradesmen will be paid, and for a time things will go on smoothly. But the dispute is by no means ended, and another salary will soon be due. As a picture in bumbledom the affairs of the Oldham Union are worthy of study, and when it is proposed to give large powers to local bodies its example should not be forgotten.

### The Hanwell Ophthalmia School.

The Hanwell Schools have been for so long associated in the public mind with ophthalmia that it is interesting to gather from a recent report of Dr. Stephenson to the Managers of the Central London School District that among the 282 patients in the ophthalmia school there was then no child from the main school at Hanwell, the last admission therefrom having been in May, 1898, and the child then admitted having been discharged after less than a month's stay. To show how tedious a complaint chronic ophthalmia or trachoma is, Dr. Stevenson gave certain figures which are worthy of quotation. By dividing the inhabitants of the school into groups according to the date of their admission, it appeared that, of those still remaining there, 6 were admitted in 1893, 4 in 1894, 3 in 1895, 13 in 1896, 40 in 1897, 153 in 1898, and 69 this year. These figures afford food for reflection to those who think that trachoma can easily be cured under proper treatment. It seems that even under the best conditions the average time occupied in the cure of the disease is about two years.

